# Consequential considerations when mapping tRNA fragments

**DOI:** 10.1186/s12859-016-0921-0

**Published:** 2016-03-10

**Authors:** Aristeidis G. Telonis, Phillipe Loher, Yohei Kirino, Isidore Rigoutsos

**Affiliations:** Computational Medicine Center, Sidney Kimmel Medical College, Thomas Jefferson University, 1020 Locust Street, Philadelphia, PA 19107 USA

**Keywords:** tRNAs, tRNA-lookalikes, tRNA fragments, tRFs, i-tRFs, 5′-tRFs, 3′-tRFs, 5′-halves, 3′-halves, RepeatMasker

## Abstract

We examine several of the choices that went into the design of tDRmapper, a recently reported tool for identifying transfer RNA (tRNA) fragments in deep sequencing data, evaluate them in the context of currently available knowledge, and discuss their potential impact on the output that the tool generates.

A recent article by Selitsky and Sethupathy [1] describes tDRmapper, a tool devised for analyzing RNA transcripts that originate from mature or precursor transfer RNAs (tRNAs). Such transcripts, commonly referred to as *tRNA fragments* or *tRFs*, have been commanding increasing attention in recent years [2–4]. In view of the repeating nature and particular sequence characteristics of both nuclear and mitochondrial tRNAs, several of tDRmapper’s stated design choices [1] will result in the labeling as tRFs of transcripts that have a significantly-high probability to originate from non-tRNA portions of the genome. This is a particularly relevant consideration given that tDRmapper was proposed as an exploratory tool. In what follows, we define as “tRNA space” the collection of 610 nuclear and 22 mitochondrial genome loci that harbor tRNA genes [5].

## Mapping to tRNA space alone, and not to the full genome, cannot unambiguously define tRNA-derived sequences

In [1], the authors present several points to support their decision for mapping to only the tRNA space and *not* to the entire genome (see Results and discussion; Step 2 of [1]). However, as we have discussed previously [6], mapping to the tRNA space alone will result in the unavoidable inclusion of sequenced reads that can be unrelated to tRNAs, thereby leading to the generation and reporting of false positives: this argument is supported by several key facts that pertain to the specifics of tRNA sequences. **First**, the human nuclear genome comprises hundreds of incomplete tRNA sequences with lengths significantly shorter than the average 72 nucleotides (nts) of the typical mature tRNA, which in turn makes them non-tRNAs. For example, for the hg19 assembly (http://www.ncbi.nlm.nih.gov/projects/genome/assembly/grc/human/index.shtml) the “tRNA” class of RepeatMasker (http://www.repeatmasker.org; Library 2014/01/31) includes 716 genomic segments, outside of tRNA space, each with length ≤ 50 nts. Because of the existing sequence similarity, tDRmapper will consider these deep-sequencing reads as tRFs even though their ambiguous genomic origin makes it distinctly probable that they do not originate from tRNAs [6, 7]. **Second**, many mature tRNAs are spliced from intron-containing precursor transcripts: deep-sequencing reads that span such tRNA exon-exon junctions cannot be claimed to be tRNA-specific without exploring the entirety of the human genome. An example of this complication is highlighted by the junction-spanning sequence ACTTCTAATTCAAA (all sequences are shown in 5′ → 3′ orientation): as implemented, tDRmapper will assign these reads to the five *genes* of ArgTCT despite the fact that this sequence has 99 exact copies in the human genome outside of tRNA space. **Third**, the presence of the non-templated “**CCA**” at the 3′ terminus of mature tRNAs creates novel CCA-ending strings that are candidate 3′-tRFs: however, these strings can also exist at multiple genomic locations that are unrelated to tRNAs. By not examining their instances outside of tRNA space, tDRmapper runs the risk of labeling as 3′-tRFs sequenced reads that are not linked to tRNAs. For example, the sequence CTCACTGGAACCT-**CCA** occurs in a single mature tRNA anticodon (GlnCTG, negative strand of chr1: 146476760-146476831, inclusive, or TRQ-CTG10-1), yet, the sequence also exists identically at 421 locations of the human genome outside of tRNA space. This 16-mer is not an isolated example. As a matter of fact, for 480 of the 632 nuclear and mitochondrial tRNAs in tRNA space, their CCA-ending 16-mers have a grand total of 2643 identical copies in the human genome outside of tRNA space: tDRmapper lets these 16-mers support a 3′-tRF even though their ambiguous origin suggests that they may not be related to tRNAs. **Fourth**, we recently showed that there are 497 loci in the human genome whose length matches that of the typical mature tRNA and whose sequences resemble, at different levels of sequence similarity, known tRNAs [5, 8]. Any deep-sequenced reads that resemble these lookalikes are of ambiguous origin, yet will be assigned by tDRmapper to currently known *bona fide* tRNA genes, which will in turn result in miscalculating a given tRNA’s potential to produce tRFs. The resulting cross-talk can potentially be very substantial: for example, 350 of the 632 nuclear and mitochondrial tRNAs share 1493 *exact* 16-mers with these 497 tRNA-lookalike sequences. For some of the tRNAs, the above four considerations will be exacerbated by tDRmapper’s allowance of mismatches and deletions. We note that strategies for overcoming localization ambiguity and controlling for potential false positive hits have already been developed and published by others [7] and by us [6].

## The inclusion of short sequenced reads will bias the generated profiles

In addition to the large number of long repeat elements, the human genome is riddled with numerous identical repetitions of sequences of length ≥ 16 nts [9]; shorter sequences appear identically at even more locations. As shown in Table 1, 92.4 % of all the 14-mers that exist in the *mature* nuclear and mitochondrial tRNAs (which include the CCA addition) also exist elsewhere on the genome. Therefore, their inclusion will result in the labeling as tRFs of 14-mers with high likelihood to originate outside of tRNA space. At the same time, as these non-tRNA 14-mers will be allowed to contribute “votes” to the accumulating profiles, they have the potential to also skew the endpoints of true tRFs that are present in the analyzed samples. In the case of 15-mers, there is still a very large fraction (79.0 %) of instances outside of tRNA space. Altogether dismissing 14-mers, and perhaps 15-mers, should reduce the rate of false positives without appreciable loss of signal. However, as we highlighted in the previous section and discussed at length in [6], for higher values of *k*, e.g., 16-mers (Table 1) or longer ones, disambiguation of the genomic origin needs to be carried out separately for each sequenced read before it can be labeled a tRF.

**Table 1:** Number of 14-/15-/16-mers from the 632 nuclearly- and mitochondrially- encoded tRNA sequences (including the non-templated CCA) with instances inside and outside of tRNA space

*k*-mer length	# of distinct *k*-mers present in nuclear and mitochondrial tRNAs	# of *k*-mers with instances exclusively inside tRNA space	# of *k*-mers with instances inside and outside of tRNA space
14	16,380	1251 (7.6 %)	15,129 (92.4 %)
15	16,733	3519 (21.0 %)	13,214 (79.0 %)
16	17,006	6972 (41.0 %)	10,034 (59.0 %)

## Associating a ‘dominant’ tRF with each tRNA sequence runs counter to the available evidence

The authors of tDRmapper purport to being able to associate a “dominant” tRF with each tRNA family, *isodecoder,* or *isoacceptor* (Results and Discussion; Step 3). This is based on their implicit assumption that the *most abundant* tRF (i.e., “dominant” per their definition) is also the *most important* one. However, as we already reported in [6], there are many examples of human tRFs that are *not* the most abundant tRFs in the analyzed samples yet can differentiate among groups of samples by gender, population origin, race, tissue, disease subtype, etc. Moreover, tDRmapper’s assigning of counts from *multiple distinct* tRFs to a *single* sequence further complicates matters, because such an aggregation of reads by tDRmapper does not acknowledge the following important fact. As we showed by analyzing hundreds of human tissue samples (see [6]), distinct tRFs that arise from the same anticodon template have uncorrelated abundances even though they can differ only slightly in sequence (the abundances of such distinct fragments are collapsed by tDRmapper into a composite ‘count’ that will suppress any available signal and skew the endpoints of the molecules that are actually present). This lack of correlation is not specific to tRNAs: indeed, it also holds true for another category of non-coding RNAs the microRNAs (miRNAs), as others [10, 11] and we reported previously [12, 13]. Specifically, in both health and disease, we have shown that different miRNA isoforms from the *same* miRNA locus can: a) have uncorrelated abundances even though they have very similar sequences [13]; b) differ greatly in their ability to distinguish between gender, population origin, race, tissue, disease subtype, etc. [12, 13]; and, c) have very distinct regulatory roles and downstream targets [13]. The numerous dependencies that tRF sequences have been shown to have on the cell/tissue type of origin, the disease type/sub-type, and the attributes of the person from whom the RNA is sourced, are evidence that does not support the hypothesis of “dominant” tRFs.

We hope that these insights prove useful in the quest to capture the tRF repertoire of samples of interest from RNA sequencing data.

**Abbreviations**

tRNA: transfer RNA; tRF: tRNA fragment; 5′-tRF: 5′ tRNA fragment; i-tRF: internal tRNA fragment; 3′-tRF: 3′ tRNA fragment; miRNA: microRNA; hg19: human genome assembly 19.

**Competing interests**

All four authors are actively involved in tRNA research and have published previously in this area. The authors declare no competing financial interests.

**Authors’ contributions**

AGT, IR and PL carried out the described analyses. IR, AGT, YK and PL wrote the commentary. All authors read and approved the final manuscript.
