# Supplementary material for: Consequential considerations when mapping tRNA fragments
Source: BMC Bioinformatics. 2016 Mar 10;17:123. doi: 10.1186/s12859-016-0921-0 (PMC4785646; doi:10.1186/s12859-016-0921-0)
Supplement: Related texts

# Sub article: Response to “Consequential Considerations When Mapping tRNA Fragments”

**Authors:** Praveen Sethupathy

**Affiliations:** Department of Genetics, School of Medicine, University of North Carolina at Chapel Hill, Chapel Hill, NC 27599 USA; Lineberger Comprehensive Cancer Center, School of Medicine, University of North Carolina at Chapel Hill, Chapel Hill, NC 27599 USA

We must first address a citation oversight. Specifically, we wish to explicitly mention here two important studies that were not cited in our original tDRmapper publication [1]. The first is Honda et al., 2015, in which the authors implicate tRNA-halves in breast and prostate cancer cell proliferation [14]. The second is Telonis et al., 2015, in which the authors develop a bioinformatic mapping scheme to establish the diversity of human tRNA-derived RNAs (tDRs) [15]. This paper was published while our tDRmapper manuscript was already under review. These two studies represent contributions to the tDR literature that merit attention.

Our primary goals with tDRmapper [1] were five-fold:

(1) To present the major challenges associated with analysis of tDRs

(2) To provide a publicly-available bioinformatic tool for annotation and quantification of tDRs from human small RNA-sequencing data

(3) To demonstrate the abundance of tDRs in several human tissues

(4) To offer new graphical visualization strategies for the analysis of tDR profiles and tDR chemical modifications

(5) To catalyze discussion among scientists in the RNA and genomics research communities about improved approaches for tDR nomenclature and quantification

In the spirit of goal #5, we welcome the commentary from Telonis et al., and we hope that it will lead to a broader conversation about the limitations of current approaches and ideas for new strategies. We provide below our responses to their two major critiques:

(1) Mapping to tRNA space instead of whole genome

Most tools in bioinformatics represent a trade-off between false negative and false positive rates. We decided in tDRmapper to reduce false negatives, while sacrificing a potential increase in false positives.

We made the decision to map to tRNA space in part because tRNA annotation in the human genome is incomplete. It remains unclear whether currently unannotated genomic regions that resemble tRNA sequences are *bona fide* tRNAs, truncated tRNAs, or unrelated to tRNAs entirely. Mapping to the whole genome would result in ambiguity about the origin of many reads. If we discard all ambiguous reads, then we run the risk of artificially lowering the true signal from annotated tRNAs. However, Telonis et al. correctly state that mapping only to annotated tRNAs in the genomic tRNA database (GtRNAdb) as opposed to the entire genome can lead to false positives, because some reads might map equally well elsewhere in the genome at incomplete-tRNA or non-tRNA loci, and these loci may be the true origin of the reads.

Consider supplemental table 6 of our recent publication in *Scientific Reports* [16], in which we list the putative tDR sequences found in human liver, as well as the coordinates of the locations to which these sequences map. The most abundant sequence, which we designate as a tRNAhalf (tRH), maps to the 5’-end of the annotated full-length Glycine tRNAs, as well as to two other locations. These other locations, which are embedded within repeat regions, harbor only incomplete Glycine tRNA sequences (it is not clear whether these regions are robustly transcribed in the liver). Therefore, the origin of the “tRH” is technically ambiguous – it could derive from the full-length Glycine tRNA or the incomplete Glycine tRNA sequences with unknown transcription status. If these reads were excluded (as suggested by Telonis et al.), a very large proportion of the tDR signal in liver tissue would be discarded, thereby running the risk of greatly inflating the false negative rate. If the reads are included (as proposed by Selitsky et al.^2^), a very large proportion of multi-mapped reads would be counted as tDRs, thereby running the risk of greatly inflating the false positive rate. In tDRmapper, we have made the decision to reduce false negative rates.

Furthermore, and perhaps more importantly, mapping to the entire genome will miss tDRs that are modified, such as by non-templated nucleotide addition (*e.g.*, 3’-terminal CCA), and therefore do not match the genomic sequence. Mapping only to annotated tRNA space eliminates these issues and further reduces the false negative rate.

We emphasize that mapping to tRNA space alone is only a short-term solution. As human genomic annotation of tRNAs improves, and as transcription status across the human genome in different tissues is clarified (via techniques such as PRO-seq), we agree that it will be important to consider alternate strategies for mapping that balance both false positive and false negative rates.

(2) Associating a single ‘dominant’ tDR with each sequence

Telonis et al. are concerned about the assignment of a single, dominant tDR to each tRNA sequence. We would like to emphasize that tDRmapper only denotes a tDR as ‘dominant’ if the reads mapping to it represent > 66 % of the reads mapping to the parent tRNA. In such cases, the ‘dominant’ tDR is at least 2-fold greater in abundance than any other tDR that may be processed from the parent tRNA. Nonetheless, we appreciate the comment from Telonis et al., because we wish to avoid what Telonis et al. referred to as the “implicit assumption that the most abundant tDR is also the most important one”. It behooves us to learn from the microRNA field, which now appreciates that non-canonical and less-abundant microRNA isoforms from the same precursor sequence can be functionally relevant and should be separately annotated and quantified. In the future, we will consider revising tDRmapper to annotate and quantify all tDRs that are above a certain user-specified threshold of read coverage.

We agree wholeheartedly with Telonis et al. that there are important considerations when analyzing tDRs and we welcome a robust discussion, as well as improved annotation of human tRNAs, and greater understanding of tDR function, in order to name and quantify tDRs in a more rational and accurate manner. We appreciate Telonis et al’s efforts to engage the discussion and contribute their important points, and we look forward to continuing to refine tDRmapper in order to address current limitations and facilitate the discovery of novel tDR biology.
